# Examining the influence of musical sophistication, cognitive performance, and social skills on the Brain Age Gap Estimate (BrainAGE)

**DOI:** 10.1007/s00429-025-03001-8

**Published:** 2025-08-11

**Authors:** Alisha D. Davis, Negin Motamed Yeganeh, Nancy Hermiston, Janet F. Werker, Lara A. Boyd, Sarah N. Kraeutner, Anja-Xiaoxing Cui

**Affiliations:** 1 Neuroplasticity, Imagery, and Motor Behaviour Laboratory, Department of Psychology, University of British Columbia, Kelowna, BC Canada; 2 Brain Behaviour Lab, Department of Physical Therapy, University of British Columbia, Vancouver, BC Canada; 3 School of Music, University of British Columbia, Vancouver, BC Canada; 4 Department of Psychology, University of British Columbia, Vancouver, BC Canada; 5 Graduate Program in Rehabilitation Sciences, University of British Columbia, Vancouver, BC Canada; 6 Department of Physical Therapy, University of British Columbia, Vancouver, BC Canada; 7 Djavad Mowafaghian Centre for Brain Health, University of British Columbia, Vancouver, BC Canada; 8 Department of Musicology, University of Vienna, Vienna, Austria; 9 Vienna Cognitive Science Hub, University of Vienna, Vienna, Austria; 10 Universität Wien, Institut für Musikwissenschaft, Spitalgasse 2, Hof 9 (Campus), 1090 Vienna, Austria

**Keywords:** Brain aging, BrainAGE, musical sophistication, cognitive ability, social skills

## Abstract

Brain age, an estimate of biological brain aging derived from neuroimaging, has been linked to cognitive and related factors. Metrics such as the Brain Age Gap Estimate (BrainAGE), depicting the discrepancy between predicted and chronological age, are commonly used to determine the influence of variables on brain aging. This study explored how cognitive ability, musical sophistication, and social skills contribute to BrainAGE in a sample of 81 healthy participants who underwent high-resolution magnetic resonance imaging and completed cognitive, musical, and social assessments. Following statistical analyses to fit the model, structural equation modelling was used to examine the influence of cognitive ability, assessed using the Delis–Kaplan Executive Function System, California Verbal Learning Test, and Wechsler Adult Intelligence Scale; musical sophistication, measured by the Goldsmiths Musical Sophistication Index; and social skills, evaluated using the Social Skills Inventory, on BrainAGE. Our findings demonstrated no significant influence of cognitive ability, musical expertise, or social skills on BrainAGE. These findings highlight the complexity of cognitive and social influences on brain age and underscore the need for further research into their interactive effects on neurobiological aging.

## Introduction

Brain aging is a natural process that occurs at varying rates across individuals, often leading to declines in cognitive functions like processing speed, memory, and fluid intelligence (Harada et al. 2013). These changes are associated with reductions in grey and white matter volumes and cortical thickness (Fjell et al. 2014; Grajauskas et al. 2019). Although brain aging is influenced by genetics, hormones, and lifestyle habits (Peters 2006), acquired skills, such as music and cognitive training, can act as neurodevelopmental agents or neuroprotective factors (Böttcher et al. 2022).

One way to measure brain aging is through the Brain Age Gap Estimate (BrainAGE), which compares an individual’s chronological age to their brain’s estimated age based on neuroimaging assessments (Ge at al. 2024). This estimate is derived using an algorithm (Centile BrainAGE) that utilizes magnetic resonance imaging (MRI)-derived subcortical volume, surface area, and cortical thickness measurements to compute an index of brain age. A positive BrainAGE score indicates accelerated aging, while a negative value suggests a younger-than-expected brain. In the following, the term BrainAGE refers to an estimated stage of brain aging derived from specific models.

Numerous daily activities have been suggested to slow brain aging. For example, musical training has been linked to enhanced cognitive abilities and protection against age-related decline, with musicians showing lower BrainAGE scores compared to non-musicians (Böttcher et al. 2022; Rogenmoser et al. 2018). However, research by Matziorinis et al. (2023) determined there was no link between musician status (i.e., amateur vs. novice) and BrainAGE, but did not include professional musicians in their sample. Together, these findings suggest that music may have a complex and not yet fully understood impact on brain aging.

In addition, strong social relationships have been shown to positively influence cognitive and brain health, reducing the risk of cognitive decline and dementia in older adults (Sommerlad et al. 2023). Social skills may also influence BrainAGE scores by promoting cognitive reserve and slowing the progression of brain aging (Ertel et al. 2011).

Lastly, cognitive ability, encompassing executive function, memory, and intelligence, among many other domains, is another key factor in brain aging. Higher cognitive ability has been associated with younger BrainAGE scores, with individuals showing greater cortical thickness and resilience to age-related changes (de Chastelaine et al. 2023). Maintaining cognitive ability over time contributes to cognitive reserve, protecting against neurodegenerative processes. Research indicates that poorer cognitive performance is linked to higher BrainAGE scores (Elliot et al. 2021; Walhovd et al. 2022). Given the importance of brain aging, we aim to explore how the aforementioned factors influence brain aging. Given that past studies have examined the individual impact of musical ability, social skills, and cognition, we assessed whether these factors interact and affect BrainAGE scores.

Therefore, the current study aimed to examine how musical ability, social skills, and cognitive ability influences the BrainAGE of participants, which indicates a change in the psychological process of brain aging. Specifically, we determined if: (1) musical sophistication positively influences brain aging demonstrated by larger, negative BrainAGE values and higher scores on the Goldsmiths Musical Sophistication Index (Gold-MSI; Müllensiefen et al. 2014), (2) having greater social skills is beneficial for brain aging, demonstrated by larger, negative BrainAGE values and greater performance (i.e., higher scores) on the social skills inventory (SSI; Riggio 2005), and (3) cognitive ability, measured by three factors (executive function, memory, and intelligence), positively influences brain aging demonstrated by larger, negative BrainAGE values and high scores on various assessment explained below.

## Methods

### Participants

Data was collected from 86 young-adult participants at the University of British Columbia (September 2019–November 2022), who provided written informed consent. Participants were recruited from the School of Music, Faculty of Arts, and varsity sports teams, and self-reported to be free of neurological or psychiatric conditions, uncorrected sensory impairments, or significant cognitive concerns. Eligible participants completed a ~ 3.5 h in-person session, including standardized behavioural assessments by a neuropsychologist and a structural MRI scan. Five participants were excluded (two due to incidental MRI findings, three for missing demographic data), resulting in a final sample of 81 (M = 20.87, SD = 3.09; 46 females: M = 20.63 (18–30), SD = 3.09; 35 males: M = 21.23 (18–32), SD = 3.13). The study was approved by the University of British Columbia Research Ethics Board.

### Assessments

Each participant completed the following assessments: Gold-MSI, SSI, Kinarm Trail Making Task (Trails; Arbuthnott and Frank 2000; Tombaugh 2004), Delis-Kaplan Executive Function System (D-KEFS; Delis et al. 2001), California Verbal Learning Test, Third Edition (CVLT-3; Delis et al. 2017), and – to avoid potential redundancy in measures and manage participant fatigue – subscales of the Wechsler Adult Intelligence Scale, Fourth Edition (WAIS-IV; Wechsler 2008; Digit Span, Matrix Reasoning, Symbol Search, and Coding subscales) and the Wechsler Memory Scale, Fourth Edition (WMS-IV; Wechsler 2009; Designs). Participants either completed the assessments first or underwent MRI, depending on scanner availability.

Summary scores were calculated for all measures, according to their respective manuals, excluding the CVLT-3 and D-KEFS, from which only the long-delay memory recall (as a measure of long-term memory) and colour-word interference test (as a measure of inhibition) subscales were utilized to avoid further redundancy in capturing cognitive abilities. Higher scores on the Gold-MSI and SSI reflect greater musical sophistication and social skills, while higher cognitive assessment scores indicate better performance, with the exception of the Kinarm Trails, where higher scores indicate impairment.

### BrainAGE and magnetic resonance imaging

BrainAGE scores were computed using the Centile BrainAGE algorithm (https://centilebrain.org/#/brainAge2), which estimates predicted age and calculates a BrainAGE score from 34 cortical and 7 subcortical measurements per hemisphere, adjusting for age and sex. Centile BrainAGE performance in our sample yielded a lower MAE (3.11 years) than typically reported with Gaser’s algorithm (≈ 5; Franke and Gaser 2019), indicating better individual prediction accuracy. However, the Centile model’s weaker correlation (*r* = 0.337) suggests Gaser’s model (R²=0.92) better explains the relationship between predicted and chronological age. High-resolution T1-weighted MRI scans were acquired on a Philips 3.0T scanner (TR = 7.4 ms, TE = 3.7 ms, flip angle = 6°, FOV = 256 mm, 160 slices, 1 mm thickness, scan time = 3.2 min) and underwent quality checking via two independent researchers. Preprocessing was performed using FreeSurfer’s recon-all function (v3.4.1; https://surfer.nmr.mgh.harvard.edu/fswiki/recon-all), which performed cortical parcellation (Desikan-Killany atlas; Desikan et al. 2006), subcortical segmentation (Aseg atlas; Fischl et al. 2002), skull stripping, and tissue segmentation and reconstruction. The aseg atlas labels and lh./rh.pial surfaces were visually inspected against the anatomical T1-weighted images to ensure accurate subcortical segmentation and cortical parcellation. Any visual issues prompted a rerun to correct imperfections. Pre-processed imaging data from 81 participants was utilized for BrainAGE computation at the participant level. Following Le et al. (2018), adjusted BrainAGE values were used in the final analysis to control for demographics.

### Data analysis

Scores from all measures were standardized (Z scores) to permit statistical testing and subsequent interpretation. Structural equation modelling (SEM) was chosen as it tests manually specified theoretical models and estimates both direct and indirect effects (Brandmaier et al. 2013). To determine which assessments meaningfully contributed to the latent variable of cognitive ability, we assessed multicollinearity. Variance inflation factors were assessed using the standard threshold of 4. With all values near 1, we confirmed the absence of multicollinearity. For means, standard deviations, and correlations between predictors, see Table 1. To address potential redundancy and unevenness, we conducted principal component, exploratory, and confirmatory factor analyses (PCA, EFA, and CFA) to empirically refine the latent cognitive ability variable, retaining only indicators with meaningful contributions. PCA and EFA identified which assessments best defined cognitive ability (Greenacre et al. 2022; Watkins et al. 2018). Separately, PCA reduced correlated predictors, while EFA assessed factor loadings to determine the minimal number of constructs. CFA then validated the fit of the retained assessments within the latent variable. Once confirmed, we proceeded with the final SEM analysis.

**Table 1:** Means, standard deviations, and correlations for all variables

	Mean (SD)	BrainAGE	D-KEFS	Trails	WAIS	SSI	G-MSI	WMS	CVLT
BrainAGE	1.09 (3.82)	----							
D-KEFS	10.88 (2.79)	0.08	----						
Trails	0.79 (0.21)	−0.09	−0.05	----					
WAIS	41.46 (7.14)	−0.05	0.42	−0.10	----				
SSI	270.53 (21.18)	0.16	0.23	0.08	−0.07	----			
Gold-MSI	82.12 (20.36)	−0.17	0.10	−0.02	0.06	0.11	----		
WMS	9.51 (4.97)	0.18	0.21	−0.09	0.21	0.16	0.02	----	
CVLT	11.27 (3.17)	−0.14	0.31	0.04	0.20	0.21	0.08	0.15	----

## Results

To create the latent variable for cognitive ability, allowing us to separately examine social skills and musical sophistication, data from the D-KEFS, Trails, WAIS-IV, CVLT-3, and WMS-IV were in the following analyses. Table 2 displays the PCA, EFA, and CFA loadings.

### PCA

The first four principal components (PCs) accounted for 88.8% of the variance, indicating that all predictor variables made a meaningful contribution to the PCs (Verma et al. 2019). Based on Kaiser’s (1960) criterion, which retains PCs with eigenvalues greater than 1, we assessed the first two PCs. PC1 was associated with executive function and intelligence. PC2 represented executive function and memory. All assessments were retained following PCA.

### EFA

Parallel analysis determined that a single factor should be retained, with factor loadings that exceeding 0.3, suggesting an association with the factor (Tavakol and Wetzel 2020). As a result, the Trails data was excluded from the final model. The fit indices indicated a very good fit to the cognitive ability factor (χ² = 1.64, *p* < 0.90, RMSR = 0.04, RMSEA = 0, 90% CI [0, 0.068], TLI = 1.328, BIC = −20.33). A final EFA with all assessments confirmed that the Gold-MSI and SSI contributed to separate factors.

### CFA

Given the conflicting guidance on whether variables with loadings < 0.3 or < 0.4 should be retained (Ondé and Alvarado 2020), the factor loading cut-off was set at 0.35. The CFA confirmed that the WAIS-IV, DKEFS, and CVLT-3 should be retained in the final model, therefore the WMS-IV was removed.

**Table 2:** PCA, EFA, and CFA standardized factor loadings for the cognitive ability variables

Variable	PC1	PC2	EFA	CFA
D-KEFS	−0.577	0.110	0.71	0.730
Trails	0.134	0.869	−0.10	N/A
WAIS-IV	−0.545	−0.079	0.59	0.573
WMS-IV	−0.400	−0.222	0.40	0.319
CVLT-3	−0.437	−0.422	0.34	0.408

### SEM

**Fig. 1:**
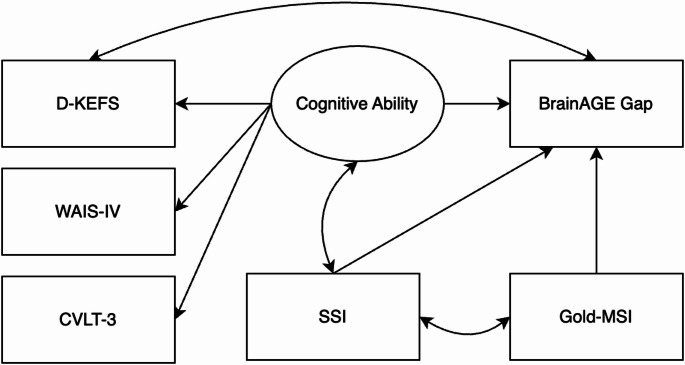
Final structural equation model (SEM)

A predefined model was developed to examine how musical sophistication, social skills, and cognitive ability influence BrainAGE, as shown in Fig. 1. Gold-MSI and SSI were treated as independent variables to assess their direct effects on BrainAGEs. Cognitive ability was represented as a latent variable, incorporating data from the D-KEFS, CVLT-3, and WAIS-IV, to evaluate its impact on BrainAGE scores. Residual covariances were added between SSI, cognitive ability, and Gold-MSI to explore shared variance among predictors. Based on prior literature and modification indices, a residual covariance between D-KEFS and BrainAGE was included to account for shared variance likely driven by overlapping neural regions not fully captured by the latent cognitive ability factor (Haas et al. 2022). The final model showed a good fit to the data (χ²(6) = 7.189, *p* = 0.304, RMSEA = 0.049 (90% CI [0.000, 0.159], *p* = 0.428 for RMSEA ≤ 0.050), CFI = 0.959, TLI = 0.898, and SRMR = 0.061).

SEM results, including factor loadings, estimates, and significance values, can be seen in Table 3. The results demonstrate that D-KEFS was a strong contributor to the latent construct, while WAIS-IV and CVLT-3 were moderate and weak contributors, respectively. There were no significant predictors for BrainAGE, demonstrating the predictors in the model did not explain a substantial amount of the variability in BrainAGE scores. Further, there were no significant interactions. The D-KEFS explained most of the residual variance in the model, however, only 8% of the variance in the BrainAGE scores was explained by the predictors, leaving 92% unexplained. We conducted exploratory models using Gold-MSI and SSI subscales, as well as individual cognitive assessments, to test whether summary scores may have obscured potential effects. However, no subscales or individual assessments emerged as significant predictors.

**Table 3:** Structural equation model (SEM) output

		Estimate	Std. Error	Z Value	*P* (>|z|)	Std.all
Latent Variables						
Cognitive ability =~	WAIS-IV	1.000				0.442
	CVLT-3	0.792	0.315	2.513	**0.012**	0.350
	D-KEFS	2.110	1.126	1.875	0.064	0.932
Regressions						
BrainAGE ~	Cog. Ability	−0.377	0.599	−0.630	0.529	−0.166
	Gold-MSI	−0.162	0.097	−1.677	0.094	−0.162
	SSI	−0.117	0.109	−1.075	0.282	−0.117
Covariances						
	Gold-MSI ~ ~ SSI	0.089	0.116	0.772	0.440	0.091
	Cog. Ability ~ ~ SSI	0.098	0.052	1.869	0.062	0.225
	D-KEFS ~ ~ BrainAGE	0.271	0.196	1.385	0.166	0.788
Variances						
	WAIS-IV	0.795	0.161	4.944	0.000	0.805
	CVLT-3	0.867	0.188	4.622	0.000	0.878
	D-KEFS	0.130	0.487	0.266	0.790	0.131
	BrainAGE	0.912	0.161	5.679	0.000	0.920
	Gold-MSI	0.988	0.135	7.340	0.000	1.000
	SSI	0.984	0.180	5.460	0.000	1.000
	Cog. Ability	0.193	0.120	1.609	0.108	1.000
R^2^						
	WAIS-IV	0.195				
	CVLT-3	0.122				
	D-KEFS	0.869				
	BrainAGE	0.080				

The estimates represent the unstandardized factor loadings (raw coefficient), std. Error represents the standard error of the estimate, and std.all represents the standardized factor loadings. Note: significant effects (p < 0.05) are bolded

## Discussion

The current study examined how cognitive ability, musical sophistication, and social skills influenced BrainAGE scores. Despite prior research suggesting that these factors may relate to brain aging (Böttcher et al. 2022; Elliot et al. 2021), our results did not yield statistically significant relationships with BrainAGE. Only 8% of the variance in BrainAGE scores were explained, indicating that additional factors likely contribute to individual differences in brain aging.

One unexamined construct was the impact of lifestyle and environmental factors. Daily habits such as physical activity, diet, sleep, and stress management have demonstrated neuroplastic effects by increasing grey and white matter volumes and elevating brain-derived neurotrophic factor, which supports neuronal survival and cognitive function (Pickersgill et al. 2022). Conversely, negative lifestyle choices like alcohol consumption and smoking are linked to reduced brain volume, cortical thinning and higher BrainAGE scores (Franke et al. 2013). Environmental influences, such as air pollution, negatively impact brain aging by inducing oxidative stress and neuroinflammation (Costa et al. 2019; Malecki et al. 2022). Further, exposure to pollutants has been linked to cognitive deficits in children and neurodegenerative processes in adults. Similarly, pesticide exposure contributes to oxidative stress, mitochondrial dysfunction, and accelerated neurodegeneration (Rodrigues et al. 2022). Socioeconomic status (SES) further influences brain aging through chronic stress, reduced cognitive performance, and poorer physical and emotional well-being (Steptoe and Zaninotto 2020). Given the established link between neurodegeneration and lifestyle and environmental factors, one would expect reductions in cortical thickness, surface area, and volume, ultimately leading to higher, positive BrainAGE scores. These effects may accelerate the BrainAGE, highlighting the critical role of lifestyle and environmental influences in shaping brain aging trajectories.

One explanation for the null effects is that BrainAGE reflects a global measure of brain aging, integrating changes in volume, surface area, and cortical thickness across multiple regions. Musical ability has been linked to structural differences in auditory and motor regions, while cognitive ability and social skills may influence prefrontal and limbic structures (Ashida and Schafer 2018; Böttcher et al. 2022; Cui et al. 2023). However, these regionally specific effects may not be strong enough to influence an aggregate metric like BrainAGE. Future research may benefit from region-specific analyses to determine whether these abilities differentially impact distinct neural structures associated with aging. Further, social skills may not fully capture the broader concept of social integration or engagement. While social participation can promote healthier behaviours, such as increased physical activity, lifestyle factors may exert independent effects on brain aging that are not directly reflected in social skill assessments. The lack of an observed relationship between cognitive ability and BrainAGE may be due to the specific assessments used. While our measures captured aspects of executive function, intelligence, and memory, they may not have provided a sufficient measure of cognitive ability. Our findings suggest that these variables do not directly influence BrainAGE but may interact with lifestyle, environmental, or genetic factors, highlighting the need for targeted neuroimaging and longitudinal studies to explore their complex role in brain aging.

A key limitation is the cross-sectional design, which prevents the determination of whether lifestyle and environment outweighed the effects of music and social skills on brain development. Further, with only 81 young participants, a fully specified SEM model would have been underpowered. Past research that found effects had larger, older samples (*n* = 140, > 60 years, Böttcher et al. 2022; *n* = 16,638, > 50 years, Ertel et al. 2011; *n* = 869, longitudinal to 45 years, Elliot et al. 2021) and therefore shows that a larger, more age diverse sample could have potentially revealed more effects. As brain development continues into the mid-20s, BrainAGE estimates at ~ 21 years may be less stable or sensitive to experience, reflecting neurodevelopment over neuroprotection and limiting generalizability. Additionally, the protective effects of musical training may only emerge with aging, when neurodegenerative processes begin – potentially explaining why significant effects are more evident in older samples (Gooding et al. 2014). Alternatively, the removal of assessments and use of summary scores may have limited the cognitive ability variable, potentially missing hypothesized effects. While ridge regression was an alternative, it does not support latent variables, meaning the cognitive ability assessments would be analysed separately. Future research should use larger samples, incorporate lifestyle and personality assessments, and consider longitudinal designs to better capture these influences.

## Conclusion

The current study aimed to investigate the effect of musical sophistication, social skills, and cognitive ability on individuals BrainAGEs. In showing that musical sophistication, social skills, and cognitive ability did not statistically influence one’s BrainAGE in a cohort of young, healthy individuals, we conclude that other factors such as lifestyle habits and environmental influences may play a larger role in impacting the brain. Future work using a larger sample should examine the influence of these factors alongside lifestyle, environmental, and personality assessments to capture a wider array of influences on BrainAGE and assess relationships between the variables.

## Data Availability

No datasets were generated or analysed during the current study.
